# Evaluation of Unconventional Supplements to the Diet of Intensively Reared Agouti (*Dasyprocta leporina*) in Trinidad, West Indies

**DOI:** 10.3390/vetsci7030108

**Published:** 2020-08-10

**Authors:** Eden Natalia John, Kegan Romelle Jones

**Affiliations:** 1 Department of Food Production (DFP), Faculty of Food and Agriculture (FFA), The University of the West Indies (UWI), St. Augustine, Trinidad and Tobago; nataliajohn0@gmail.com; 2 Department of Basic Veterinary Sciences (DBVS), School of Veterinary Medicine (SVM), Faculty of Medical Sciences, The University of the West Indies (UWI), Mt. Hope, Trinidad and Tobago

**Keywords:** *Trichanthera gigantica*, *Oryza glaberrima*, Moruga hill rice, agouti, rabbit ration, proximate analysis

## Abstract

A feed trial was carried out to evaluate potential unconventional feed resources such as *Trichanthera gigantica* and moruga hill rice (*Oryza glaberrima*) as a partial supplementation to the diet of adult male agoutis (*Dasyprocta leporina*). Supplemental feeding of the agouti will decrease feeding cost to produce this animal and aid in sustainable agricultural practices. Male agoutis were used as this physiological state was the easiest to attain as well as male animals are generally used for meat production. The trial consisted of 16 male agoutis that were allocated into four dietary treatments using a completely randomized experimental design. Four diet treatments were used in the experiment; treatment 1 (T1) was the positive control which consisted of rabbit ration whilst treatment 2 (T2) to 4 (T4) had different ratios of *O. glaberrima*, *Trichanthera gigantica* and Rabbit Ration. The feeding trial had 8-week duration. T2 consisted of 5% *T. gigantica*, 35% *O. glaberrima* supplemented for rabbit ration. T3 had 10% *T. gigantica* and 40% *O. glaberrima* supplemented for rabbit ration. T4 consisted of 15% *Trichanthera* and 45% *O. glaberrima* supplemented for rabbit ration in the diet. Proximate analysis showed that Dry Matter (DM) and Ash was highest in T4 and lowest in T2. Whilst ether extract (EE) and crude protein (CP) were highest in T2 and lowest in T4. Crude fiber (CF) was highest in T3 and lowest in T2. The weights of the agoutis at the start of the experiment (2595 g–2971 g) were not significantly different to their final weight (2469–2762 g) (*p* > 0.05). There was a significant difference seen between treatments groups and weeks of the experiment (*p* < 0.05). There was no significant difference in the interactions between treatment and weeks (*p* > 0.05). T1 and T2 were not significantly different (*p* > 0.05) with respect to average daily gain (−0.98 g/d, −1.61 g/d) and weight loss (55 g, 90 g). T2 can be used as an alternative feed source than rabbit ration (control diet) for adult male agoutis. As the final body mass of the male agoutis did not change with the inclusion of 40% *Trichanthera* and Moruga hill rice, this substitution can be used to maintain male agoutis before slaughter. However, higher amounts of supplements may be detrimental to this animal.

## 1. Introduction

The agouti (*Dasyprocta leporina*) is a neo-tropical rodent that can be found in forested and wooded areas of the Caribbean Antilles, Mexico, Central and South America [1]. They have small home ranges and are essential as seed dispersers [2,3]. They are monogastric mammals that practice caecotrophy and coprophagy [4]. Wild agoutis have been reported to consume pulp from fruits that are plentiful and the seeds when fruits are scarce [5]. 

The Agouti (*D. leporina*) has been greatly valued for its meat and, therefore, has the potential for providing economic and social gain to inhabitants of the Neo-tropical region [6]. In the Neo-tropics it has been utilized as a source of meat protein and in medicinal therapies. [7,8]. To satisfy the increasing demand for this wild animal protein without threats to endangering the agouti, the development of an intensive production system for the commercial production of agouti in captivity has been advised [9]. 

The diet of the agouti in the wild and in captivity has been described as being omnivorous. These animals have been found eating animal matter such as bruchid larvae [10,11], carrion [12], eggs [13], insects, and in some cases young agouti (cannibalism) [14]. The agouti has a simple stomach with a functional caecum. It practices both caecotrophy and coprophagy [15]. 

Broken rice has been fed to monogastric animals such as pigs at various levels as a substitute for maize without deleterious effects [16,17]. Broken rice is composed of the germ, chipped and broken kernels that have a low fiber content and high energy value making it a valued energy feed [18]. In rabbits, broken rice was incorporated into a balanced diet as high as 20% [19,20,21]. Moruga hill rice is locally produced for human consumption. The by-products of this rice can be used as livestock feed. However, this species of rice has not been recorded in the literature for feeding animals. The use of the by-products for this species of rice decreases the feed cost for the farmer, increases the farmers’ sustainability, as well as promotes integrated farm animal practices.

*Trichanthera* (*T. gigantica*) is forage that can be harvested every 3 months with a yield of 17 t/ha/year of fresh matter. Protein found in the *Trichanthera* leaves has a good balance of amino acids, and based on work done, there are a few secondary plant compounds [22]. It has been used as a foliage supplement in pigs [23,24,25,26], rabbits [22,27] and guinea pigs [22]. *Trichantera* is a useful forage to be used as animal feed. As stated above, it can produce large yields of fresh forage with high levels of protein and low toxic compounds. This forage can be grown of various soils and produces highly nutritious feed. This is because this forage has the ability to fix nitrogen and increase the fertility of the soil. 

To the authors’ knowledge, this is the first time the use of by-product feeds are investigated in the performance of the agouti. Supplemental feeding of the agouti will decrease feeding cost to produce this animal and aid in sustainable agricultural practices. Male agoutis were used as this physiological state was the easiest to attain as well as male animals are generally used for meat production. 

The objective of the experiment was to investigate the effects on the live weight that feeding broken rice and hulls from the Moruga Hill Rice (*O. glaberrima*) and *Trichanthera* (*T. gigantica*) at different ratios will have on the agouti.

## 2. Materials and Methods

### 2.1. Ethical Approval

All applicable international, national, and/or institutional guidelines for the care and use of animals were followed. The research site has been overseen by veterinarians to ensure animals are kept healthy. Field and laboratory protocols were approved by the Ethics Committee of the University of the West Indies, Faculty of Food and Agriculture, University of the West Indies, St. Augustine campus (ref no. CEC346/05/18). 

### 2.2. Animal Management and Husbandry

This experiment had a preliminary period of 1 week, which was followed by a data-recording spanning a period of 8 weeks at the University Field Station (UFS). Proper husbandry procedures were done during this experiment. Sixteen adult males with an average weight of 2500–3000 g were used. They were removed from a grow-out floor pen and their initial weights were taken, after which they were placed into individual cages. Before the experiment, these animals were fed local fruits and rabbit pellets (Mastermix Ltd.^®^) ad libitum. The animals were fed daily with old feed being discarded and replaced with fresh feed. 

### 2.3. Experimental Design and Feeding Trials

The experimental design was completely randomized with four treatments and four subjects per treatment. Treatment 1 (T1) (Control) was a 100% Commercial Rabbit Ration Master Mix. Treatment 2 (T2) consisted of a mixture of 5% *Trichanthera*, 35% Moruga Hill Rice and 60% Rabbit Ration. Treatment 3 (T3) was a 10% *Trichanthera*, 40% Moruga Hill Rice and 50% Rabbit Ration. Treatment 4 (T4) had 15% *Trichanthera*, 45% Moruga Hill Rice and 40% Rabbit Ration. The ratios of Moruga hill rice and *Trichantera* were chosen based on previous reports in rabbits. A maximum inclusion of 15% *Trichantera* and 45% MHR was based on work done in rabbits.

The chemical composition of the feed ingredients and diets is given in Table 1. The composition content of *Trichanthera*, with the exception of EE, was higher than both Moruga Hill rice and rabbit ration. The EE content of rabbit ration was higher than that of Moruga hill rice and *Trichanthera*. The experimental diets, T2, had a higher composition content of DM, EE and CP but the lowest Ash and CF when compared to dietary Treatments 3 and 4. Treatment 3 and Treatment 4 had similar composition contents. The Ash and CF contents increased, whereas the EE and CP content decreased with the increasing levels of Moruga hill rice and *Trichanthera* in the diet (Table 1).

### 2.4. Feed Making Process of Trichanthera and the Broken Rice

The *Trichanthera* leaves were harvested from the field at the University Field Station (UFS), St. Augustine, Saint George, Trinidad. After harvesting, it was taken to the Feed Mill building where it was laid out on feed bags and wire mesh to air dry. The semi-dried leaves were then transported to the Food Production Lab (FPL) on the University of the West Indies (UWI), St. Augustine campus, where the leaves were packed into the oven trays and place into the ovens at 60 °C for 48 h. After 48 h, the dried leaves were taken out and emptied into feed bags and taken to the Greenhouse located on campus. The Thomas-Wiley laboratory Mill Model 4^®^, Orlando, FL, USA was used to mill the dry *Trichanthera* leaves into a powdered form. The Thomas–Wiley laboratory Mill Model 4^®^ was equipped with a 2 mm sieve to ensure that a constant particle size was obtained. 

The dried leaves in the feed bag were compressed until the leaves were crumbled, and then it was emptied into a bucket, so it was easier to scoop into the top of the mill. The pulverized leaves that collected in the jar were emptied into ziploc bags and stored in the FPL until they were used. The Moruga Hill Rice was taken directly to the Greenhouse where they were emptied into a bucket to be milled with the same equipment used for the *Trichanthera*. As the jar filled with the pulverized rice hulls, they were emptied into ziploc bags that prevented moisture from entering and stored in the FPL until being used. The dried ingredients were placed into a pelletizing machine at the rated stated above and 2 × 2 pellets were made. The feed was prepared weekly for the entire trial. The feed pellets were prepared to ensure that each feed treatment was of similar size, as pellet size affects feed intake.

### 2.5. Measurements and Laboratory Analysis

Individual animals were removed from their cages, placed into pre-weighed feed bags and their weights were recorded at the beginning of each week for the period of October 2019 to February 2020. Feed was measured out and given to each agouti after animals were weighed. Proximate analysis was done on the feed treatments, *Trichanthera* leaves, Moruga Hill Rice, and Rabbit Ration to determine the following: Dry Matter (DM), Ash, Ether Extract (EE), Crude Fiber (CF) and Crude Protein (CP) according to AOAC (1990) [28].

### 2.6. Data Analysis

Data was analyzed using a completely randomized design with the different diet treatments against time using the SPSS (20) one-way ANOVA. Repeated measures analysis was performed to test the difference between treatment groups. A significance level of *p* < 0.05 was used throughout the experiment. 

## 3. Results

This feed trial consisted of four treatment groups each with varying percentage of feed supplementation. The control diet and the diet that had 40% supplementation (5% T, 35 MHR) showed the least weight lost in comparison to the other treatments. Treatment 4 started with the lowest initial body mass but had intermediate weight loss. The initial week’s mean value was significantly different when compared to weeks 1 to 7 (*p* < 0.05) (Table 2). There were significant differences between the treatment groups (*p* < 0.05) and no significant difference in the interaction between weeks and treatment (*p* > 0.05). Weeks 0 and 8 showed no significant difference in live weight of the animal (*p* > 0.05).

**Figure 1:**
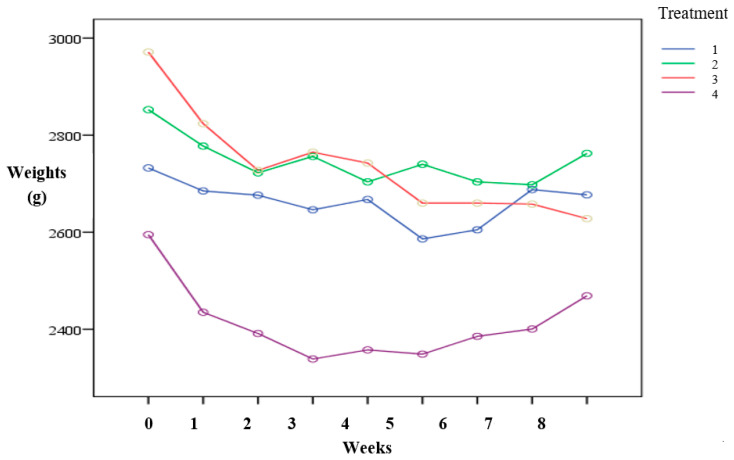
The average weights of each treatment over the 8-week period.

The control group and the 5% *Trichantera* 35% MHR treatment were not statistically different (*p* > 0.05). These groups showed the best results of the feed trial. Animals within these groups lost minimal weight (2.01%, 3.16%) throught the trial period. Animals within treatment 4 had intermediated results with an average weight loss of 4.86%. Animals present in treatment 3 had the least favorable results amongst all treatment groups. Animals present in treatment 3 had an average weight loss of 11.54% in the feed trial (Table 3, Figure 1). Supplimenting with 5% *Trichantera* and 35% moruga hill rice showed no significant difference in final weight when compared to the control diet. This shows that the agouti can be supplimented with unconventional feed resources for intensive production purposes.

## 4. Discussion

In this feeding trial, the Moruga hill rice gave higher values for ether extract (4.31%), crude fibre (1.88%) and ash content (1.8%), when compared to other reports [29]. The differences can be attributed to the fact that Heuzé et al. [29] used broken rice and not Moruga hill rice, which are two different plant species. The dry matter (92.9%), crude protein (18.25%) and ash (23.50%) content of *Trichanthera* in this experiment were higher than the of Heuzé et al. [30]. Heuzé et al. [30] obtained 91.0% dry matter, 16.9% crude protein and 20.1% ash. Crude fibre (13.3%) and ether extract (1.49%) content were lower than that reported by Heuzé et al. [30]. Variations in values can be due to the environment (temperature, sunlight, soil composition) in which the forage was grown as well as differences in the processing of the forage. The higher values of ether extract (4.31%) found in Moruga hill rice and crude protein (18.25%) in the *Trichantera* made this mixture a suitable supplemental feed for the agouti. It was seen that this supplement can be utilized in the diet of the agouti at a maximum limit of 40% without affecting the animal’s final body weight.

This is the first experimental feeding trial for *Dasyprocta leporina* where these animals can be supplemented with 40% by-product feed. Increasing proportions of *T. gigantica* in the feed also resulted in increased weight loss. Increasing the proportion of *Trichantera* in the diet increased the fibre present in the diet. The agouti is monogastric hindgut fermenter [15]. One possibility is that the increase in fibre decreased the digestibility of the feedstuff resulting in decreased weight gain. The agouti being a hindgut fermenter has the ability to digest forages. However, in this experiment, increasing the content of fibre caused a decline in weight loss. In this experiment, the extent of coprophagy and caecotrophs were not evaluated. The cages did not allow the animal to practice coprophagy and may have decreased these animals’ ability to utilize fibre. The second possibility is that *Trichanthera* has non-nutritional factors such as tannins that can have a negative effect on digestibility and feed intake in monogastric animals [31,32]. Tannin affects the palatability of *Trichanthera* because of its bitter taste leading to a reduced feed intake [33]. Treatment 4 had the highest amount of *Trichanthera* at 15% (150 g/kg). Treatment 2, which had the lowest proportion of tannins in the diet, showed no statistical difference in live weight when compared to the control diet (0% tannins). This shows that the maximum level of *Trichanthera* that can be used as a supplement in feeding the agouti is less than 10%. 

Data on diet supplementation with *T. gigantica* and broken rice with the effects of their inclusion in the diets of mono-gastric mammals is limited. *Trichanthera* was used in the diets of domestic rabbits (*Oryctolagus*) at the following rates 90, 180 and 270 g/kg DM [26]. The rabbits in that experiment showed no deleterious effects with high dietary levels of *Trichanthera*. However, in this experiment, *Trichanthera* was fed at 50, 100 and 150 g/kg DM with major weight losses occurring at the higher concentrations. Broken rice was used to feed rabbits at the highest inclusion rate of 40% to replace maize without any observable problems [34].

The inclusion of rice in the diet of pigs improved the digestibility and absorption of energy better than corn [35]. Pigs and rats fed the same diets of a broken rice and corn (50/50) had a reduction in feed intake and an increase in feed conversion with the broken rice diet [36]. However, in our experiment, the animals supplemented with broken MHR in conjunction with other supplemental ingredients had decreased weight gain and the feed conversion was not reported. The use of unconventional feed resources for non-domesticated species such as the agouti is ideal for sustainable agriculture. In this time of climate change and global pandemics, wild animals reared on feed by-products can improve food security for persons living in rural communities in the Neo-tropics.

## 5. Conclusions

The information from the above experiment has shown that adult male agoutis can be supplemented with Moruga hill rice and *Trichantera* (maximum 40%) without any changes to the final weight. As such, this diet can be fed to adult male agoutis before slaughter. However, higher amounts of supplements may be detrimental to this animal.

## Figures and Tables

**Table 1:** Proximate analysis of the feed ingredients and diet treatments used.

Feed	Composition % DM				
	DM (%)	Ash (%)	EE (%)	CF (%)	CP (%)
Ingredients					
Moruga Hill Rice	87.44	1.80	4.31	1.88	8.93
*Trichanthera*	92.90	23.50	1.49	13.3	18.25
T1-Rabbit Ration 100%	88.39	6.92	5.49	5.54	17.03
Dietary Treatments					
T2 (5% T, 35% MHR, 60% RR)	93.49	5.93	3.45	4.47	14.87
T3 (10% T, 40% MHR, 50% RR)	94.19	7.00	2.98	5.14	13.71
T4 (15% T, 45% MHR, 40% RR)	94.38	7.16	2.59	5.13	13.38

**Table 2:** Body mass of Agouti in various feed treatments groups over an 8-week period.

Week	Control (Mean ± SD)	T2 (Mean ± SD)	T3 (Mean ± SD)	T4 (Mean ± SD)
0	2732.50 ± 121.826 ^a^	2852.50 ± 157.612 ^a^	2971.25 ± 281.140 ^a^	2595.00 ± 285.628 ^a^
1	2685.00 ± 110.454 ^b^	2777.50 ± 161.478 ^b^	2823.75 ± 260.428 ^b^	2435.00 ± 244.643 ^b^
2	2676.25 ± 166.652 ^b^	2722.50 ± 210.970 ^b^	2727.50 ± 274.848 ^b^	2391.25 ± 226.325 ^b^
3	2646.25 ± 131.236 ^b^	2756.25 ± 236.198 ^b^	2765.00 ± 266.396 ^b^	2338.75 ± 227.353 ^b^
4	2667.50 ± 126.524 ^b^	2703.75 ± 167.102 ^b^	2742.50 ± 245.849 ^b^	2357.50 ± 235.885 ^b^
5	2586.25 ± 82.903 ^b^	2740.00 ± 224.165 ^b^	2660.00 ± 209.921 ^b^	2348.75 ± 245.200 ^b^
6	2605.00 ± 60.690 ^b^	2703.75 ± 212.970 ^b^	2660.00 ± 216.372 ^b^	2385.50 ± 274.149 ^b^
7	2688.00 ± 100.731 ^b^	2697.75 ± 193.222 ^b^	2658.00 ± 224.006 ^b^	2400.50 ± 289.535 ^b^
8	2677.00 ± 139.107 ^a^	2762.50 ± 241.199 ^a^	2628.00 ± 286.901 ^a^	2469.00 ± 333.269 ^a^

^a,b^ Means in the same column that had different subscripts are significantly different (*p* < 0.05). Treatment 1–100% Rabbit Ration, Treatment 2–5% *Trichanthera*, 35% Moruga Hill Rice and 60% Rabbit Ration, Treatment 3–10% *Trichanthera*, 40% Moruga Hill Rice and 50% Rabbit Ration, Treatment 4–15% *Trichanthera* and 45% Moruga Hill Rice and 40% Rabbit Ration.

**Table 3:** Live weight of the agouti in different experimental feed groups.

	Control	T_2_	T_3_	T_4_	*p* Value
Initial Live weight (g)	2732	2852	2971	2595	0.32
Final Weight (g)	2677	2762	2628	2469	0.12
Average daily gain (g/ day)	−0.98 ^a^	−1.61 ^a^	−6.13 ^b^	−12.94 ^c^	0.04
Weight loss (g)	55 ^a^	90 ^a^	343 ^b^	126 ^c^	0.02
Weight loss (%)	2.01 ^a^	3.16 ^a^	11.54 ^c^	4.86 ^b^	0.03

^a,b,c^ Means in the same row that had different subscripts are significantly different (*p* < 0.05).
